# Metastatic colon cancer cell populations contain more cancer stem-like cells with a higher susceptibility to natural killer cell-mediated lysis compared with primary colon cancer cells

**DOI:** 10.3892/ol.2015.2918

**Published:** 2015-01-29

**Authors:** GA RIM KIM, GA-HEE HA, JAE-HO BAE, SAE-OCK OH, SUN-HEE KIM, CHI-DUG KANG

**Affiliations:** 1 Department of Biochemistry, Pusan National University School of Medicine, Yangsan, Gyeongsangnam-do 626-870, Republic of Korea; 2 Department of Anatomy, Pusan National University School of Medicine, Yangsan, Gyeongsangnam-do 626-870, Republic of Korea

**Keywords:** metastatic colon cancer cells, clonogenicity, DR5, NKG2D ligands, natural killer cell-mediated lysis

## Abstract

In the present study, the soft agar clonogenicity and the susceptibility of clonogenic cancer cells to natural killer (NK) cells were compared between primary colon cancer cells (KM12C) and metastatic colon cancer cells (KM12L4a and KM12SM) to determine whether the metastatic cancer cells consisted of more cancer stem-like cells and were resistant to NK cell-mediated lysis. The majority of colon cancer cells were positive for putative cancer stem cell markers, including CD44, CD133 and EpCAM, with the exception of KM12C cells, of which only ~55% were positive for CD133. In addition, the expression levels of sex determining region Y-box 2, Nanog and octamer-binding transcription factor 4, which are essential for maintaining self-renewal, were higher in KM12L4a and KM12SM compared with that in KM12C cells. Consistently, an increased clonogenicity of KM12L4a and KM12SM compared with KM12C cells in soft agar was observed. The expression levels of NKG2D ligands, including major histocompatibility complex class I polypeptide-related sequence A/B and UL16 binding protein 2, and of death receptor 5 were significantly higher in KM12L4a and KM12SM than in KM12C cells. Furthermore, the results indicated an increased susceptibility of KM12L4a and KM12SM to NK cell-mediated cytotoxicity in comparison with KM12C cells. These results indicated that metastatic colon cancer cell populations may consist of more cancer stem-like cells, and have greater susceptibility to NK cell-mediated lysis compared with that of primary colon cancers.

## Introduction

Whilst patients with a well-confined primary tumor may be treated by surgical removal of the tumor, metastatic diseases are frequently incurable, and are the primary cause of cancer morbidity and mortality (1,2). It is estimated that metastasis is responsible for ≤90% of mortalities from solid cancers (1,2). Colorectal cancer is one of the major causes of cancer-related mortality worldwide, despite various combinations of current treatments, including surgery, chemotherapy, and/or radiation. This failure is primarily due to the resistance to existing anticancer therapies for metastatic tumor cells, which may be originate from cancer stem cells (3). Therefore, it is necessary to understand the characteristics of metastatic cancers in order to develop new therapeutic approaches.

Cancer stem cells (CSCs) are thought to be responsible for cancer initiation, progression, metastasis, recurrence and drug resistance (4). As a source of migratory/invasive cells in primary tumors, CSCs appear to be essential for the formation of distant metastases (3). As CSCs are naturally resistant to a variety of therapeutic insults (5), metastatic cells may also be resistant to therapy. Indeed, resistance to apoptosis appears to be an important component of metastasis. It has been demonstrated that tumor cells with resistance to apoptosis are more likely to successfully establish metastases (6,7), and the resistance to apoptosis is increased during metastatic dissemination of colon cancer (8). Therefore, highly metastatic tumors may comprise a greater proportion of CSCs compared with primary tumors.

Cancer immunotherapies have been considered as a potential approach to eliminate CSCs, due to the reported role of immune effector cells, including CD8+ T cells and natural killer (NK) cells, in the prevention of tumor formation (9,10). Little has been established with regard to the susceptibility of CSCs to NK cell-mediated lysis. NK cell-mediated target cell lysis and apoptosis is a cell-contact-dependent process, which acts via the release of cytotoxic granules containing perforin and granzymes, and death receptor (DR) pathways, including TNF-related apoptosis-inducing ligand (TRAIL) receptors or Fas (11,12); this may be regulated by a balance between activatory and inhibitory signals, and cell death signals (13,14).

In the present study, the clonogenicity in soft agar, and susceptibility to NK cells of clonogenic colon cancer cells was compared between the colorectal cancer cell line KM12C and its highly metastatic sublines, KM12SM and KM12L4A.

## Materials and methods

### Cell lines and reagents

The poorly metastatic KM12C cells (University of Texas MD Anderson Cancer Center, Houston, TX, USA), which were established from a primary colorectal carcinoma classified as Dukes, and the highly metastatic KM12SM and KM12L4A cells (both obtained from University of Texas MD Anderson Cancer Center), which were derived from KM12C cells (15), were maintained at 37°C in humidified atmosphere of 5% CO_2_, in Dulbecco’s modified Eagle’s medium (Gibco, Grand Island, NY, USA) supplemented with 10% fetal bovine serum (FBS), 2mM L-glutamine, 100 units/ml penicillin and 100 μg/ml streptomycin. NK-92 cells were maintained in α-minimum essential medium (Lonza, Walkersville, MD, USA) containing 12.5% FBS, 12.5% horse serum (Gibco), 2 mM L-glutamine, 0.1 mM 2-mercaptoethanol, and 400 U/ml recombinant human interleukin-2 (Novartis, Surrey, UK). Anti-major histocompatibility complex (MHC) class I polypeptide-related sequence A/B (MICA/B; cat. no. MAB1300) and anti-UL16 binding protein (ULBP)1/2/3 (cat. no’s. MAB1380, MAB1298 and MAB1517) antibodies were purchased from R&D Systems, Inc. (Shanghai, China). Allophycocyanin (APC)-conjugated anti-mouse IgG, and fluorescein isothiocyanate (FITC)-conjugated anti-CD44 and anti-epithelial cell adhesion molecule(EpCAM)-PerCP Cy5.5 antibodies were purchased from BD Biosciences (Franklin Lakes, NJ, USA). Phycoerythrin (PE)-conjugated anti-CD133 antibody was purchased from Miltenyi Biotec Inc. (Cambridge, MA, USA) and mouse monoclonal antibodies against TRAIL-R1 and R2 were purchased from Enzo Life Sciences (Farmingdale, NY, USA).

### Flow cytometric analysis

Flow cytometry was conducted to evaluate the surface expression levels of CD133, CD44, EpCAM and NKG2D ligands. Cells were harvested, washed twice with cold phosphate buffered saline, and subsequently incubated with the appropriate antibodies, according to the manufacturer’s instructions, for 30 min at 4°C in the dark: For the evaluation of CSC marker expression, cells were stained with PE-conjugated anti-CD133, FITC-conjugated anti-CD44 and PerCP-conjugated EpCAM; to assess the expression of NKG2D ligands and TRAIL receptors, cells were stained with anti-MICA/B, ULBP1, ULBP2 and ULBP3 primary antibodies, and anti-DR4 and DR5 primary antibodies, followed by incubation with APC-conjugated secondary antibody. Flow cytometric analysis was performed using FACSCalibur or FACSCanto II (BD Biosciences), and analyzed with CellQuest version 3.1 software (BD Biosciences) or BD FACSDiva version 2.1.6 software (BD Biosciences), respectively. Normalized mean fluorescence intensity (MFI) was calculated by subtracting the MFI of the isotype control from the MFI of the specific antibody (16,17).

### RNA isolation and reverse transcription quantitative polymerase chain reaction (PCR)

Total RNA was isolated using the RNeasy mini kit (Qiagen, Valencia, CA, USA) and reverse transcribed using M-MLV Reverse Transcriptase (Promega Corporation, Madison, WI, USA) and Random Primers (Takara Biotechnology Co., Ltd., Dalian, China). Real time-PCR was conducted with PowerSybr Green Master Mix (Applied Biosystems, CA, USA) according to the manufacturer’s instructions, using specific primers for sex determining region Y-box 2 (SOX-2; forward, 5′-GAG ACC GAG CTG AAG CCG CC-3′; reverse, 5′-GCC CAG GCG CTT GCT GAT CT-3′), octamer-binding transcription factor 4 (OCT-4; forward, 5′-AAC TCC GAT GGG GCC TCC CC-3′; reverse, 5′-CTC GAG CCC AAG CTG CTG GG-3′), NANOG (forward, 5′-AGC CAG AAG GCC TCA GCA CC-3′; reverse, 5′-GGC CTT CCC CAG CAG CTT CC-3′) and β-actin (forward, 5′-CAG AGC AAG AGA GGC ATC CT-3′; reverse, 5′-TTG AAG GTC TCA AAC ATG AT-3′). The relative transcript copy number per sample was normalized to β-actin and calculated using the 2[−ΔΔC(T)] method (18).

### Soft agar colony formation assay

The soft agar colony formation assay was conducted using 96-well culture plates. Each well was coated with 50 μl bottom agar mixture in medium containing 10% FBS and 0.5% agar (Sigma-Aldrich, St. Louis, MO, USA). The bottom layer was overlaid with 50 μl top agar mixture in medium containing 10% FBS, 0.35% agar, and 300 cells (KM12C, KM12SM or KM12l4A). Following a two-week incubation at 37°C in a humidified atmosphere of 5% CO_2_, colonies larger than 100 μm in diameter were counted with an inverted microscope (Olympus CKX41) equipped with a camera (Olympus DP72) and image analyzer (DP2-BSW). Colony forming efficiency was defined as (colonies per well)/(cells seeded per well). For NK92-mediated cytotoxicity, a soft agar colony formation assay was performed following co-culture of the target cells with NK-92 cells for four hours at various effector to target ratios (0:1, 0.1:1, 1:1, 5:1 and 10:1).

### Statistical analysis

Data are presented as the mean ± standard errors. For comparison of groups, an unpaired Student’s *t*-test was performed. P<0.05 was considered to indicate a statistically significant difference in all experiments.

## Results

### Comparison of CSC markers between primary and metastatic colon cancer cells

**Figure 1:**
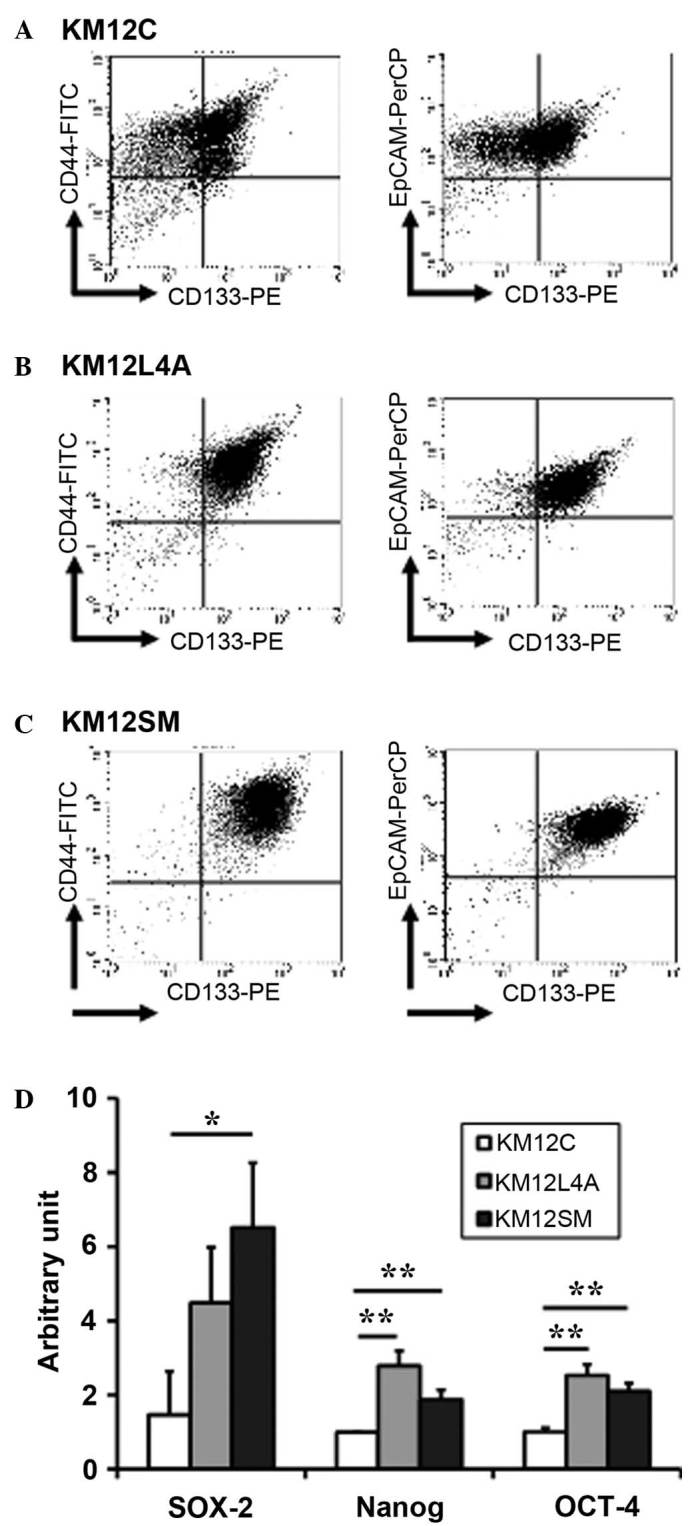
Comparison of expression levels of putative colon CSC markers and stemness-related genes between the primary KM12C cells and the highly metastatic KM12L4A and KM12SM cells. Flow cytometric analysis of putative colon CSC markers was conducted in (A) KM12C, (B) KM12L4A and (C) KM12SM cells. (D) Expression of SOX-2, Nanog, and OCT-4 in three sublines. Data are presented as the mean ± standard error of three independent experiments, and were analyzed using a Student’s *t*-test (^*^P<0.05; ^**^P<0.01). CSC, cancer stem cell; SOX-2, sex determining region Y-box 2; OCT-4, octamer-binding transcription factor 4.

To examine whether highly metastatic cancer cell populations contain a greater proportion of CSCs, the surface expression of several putative colon CSC markers was determined in KM12C (Fig. 1A), KM12L4A (Fig. 1B) and KM12SM (Fig. 1C) cells. The majority of KM12L4A and KM12SM cells were positive for CD44 (96.89 and 99.19%, respectively), CD133 (91.9 and 98.29%, respectively) and EpCAM (97.39 and 98.81%, respectively). Although a high proportion of KM12C cells were also positive for CD44 and EpCAM, CD133 expression was only detected in ~55% of these cells. In addition, the mRNA levels of SOX-2, NANOG and OCT-4, which are essential for maintaining self-renewal (19), were higher in KM12L4a and KM12SM compared with that in KM12C cells (Fig. 1D). These results indicate that highly metastatic cancer cells may have more cancer stem-like cells than primary cancer cells.

### Clonogenicity of metastatic colon cancer cells

**Figure 2:**
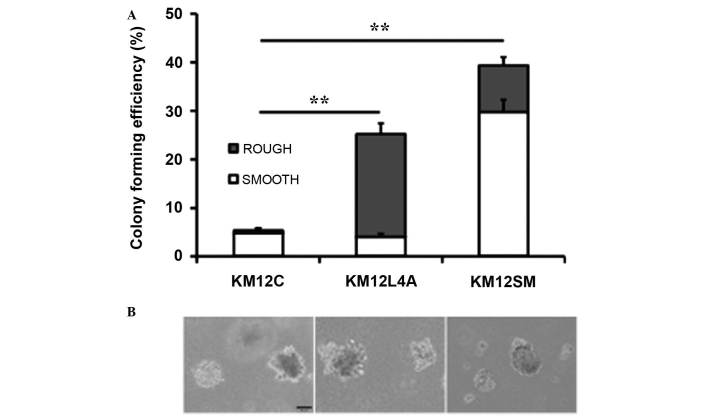
Comparison between primary KM12C cells and highly metastatic KM12L4A and KM12SM cells with regard to (A) colony forming efficiency and (B) colony phenotypes in soft agar. Colony forming efficiency was defined as (colonies per well)/(cells seeded per well). Data, presented as mean ± standard error of three independent experiments, were analyzed by a Student’s *t*-test (^*^P<0.05; ^**^P<0.01). Microscopic images show representative colonies for each cell type (scale bar, 100 μm).

As colony-formation in soft agar is thought to be characteristic of cancer stem cells (20), the clonogenicity of KM12C cells and their highly metastatic sublines was investigated. The clonogenicity of KM12C, KM12L4A and KM12SM cells was approximately 5.4, 25.2 and 39.3%, respectively (Fig. 2). These results were consistent with the levels of putative cancer stem cell markers and stemness genes measured by flow cytomtery. However, colony morphology differed between these sublines. The majority of KM12C colonies possessed a smooth surface with a spheroid appearance whilst KM12L4A and KM12SM colonies cells exhibited mixed morphologies. KM12L4A cell colonies had predominantly rough surfaces, with the appearance of spherical cellular aggregates, and KM12SM cells colonies had predominantly smooth surfaces.

### Comparison of NKG2D ligands and death receptors between primary and metastatic colon cancer cells

**Figure 3:**
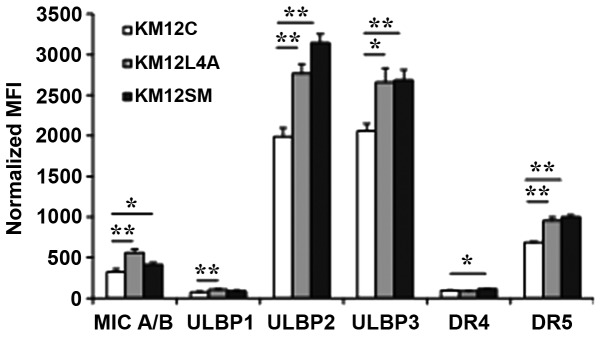
Comparison of expression of natural killer cell NKG2D ligands and TNF-related apoptosis-inducing ligand receptors between the primary KM12C cells and the highly metastatic KM12L4A and KM12SM cells. Normalized MFI was calculated as specific staining minus isotype control staining. Data, presented as mean ± standard error of three independent experiments, were analyzed by a Student’s *t*-test (^*^P<0.05, ^**^P<0.01). MFI, mean fluorescence intensity; MIC A/B, major histocompatibility complex class I polypeptide-related sequence A/B; ULBP, UL16 binding protein; DR, death receptor.

The susceptibility of metastatic cancer cells to NK cell-mediated lysis was investigated by comparing the levels of activating NKG2D ligands and of TRAIL receptors (DR4 and DR5) between KM12C, KM12L4A and KM12SM cells (Fig. 3). The surface expression levels of MICA/B, and ULBP2 and 3 were significantly higher in KM12L4A (P=0.00175, 0.00116 and 0.01597, respectively) and KM12SM cells (P=0.02149, 0.00019 and 0.00258) compared with KM12C cells. For ULBP1, the level of expression was higher in KM12L4A cells than in KM12C cells. The level of DR5 was also significantly higher in KM12L4A and KM12SM cells than in KM12C cells. For DR4, the level was higher in KM12SM cells (P=0.00054) than in KM12C cells. These data indicate that the levels of NKG2D ligands and death receptors were generally higher in the metastatic KM12L4A and KM12SM cells than in the primary KM12C cells, suggesting that metastatic colon cancer cells may not be resistant to NK cell-mediated lysis.

### Susceptibility of metastatic colon cancer cells to NK92 cells

**Figure 4:**
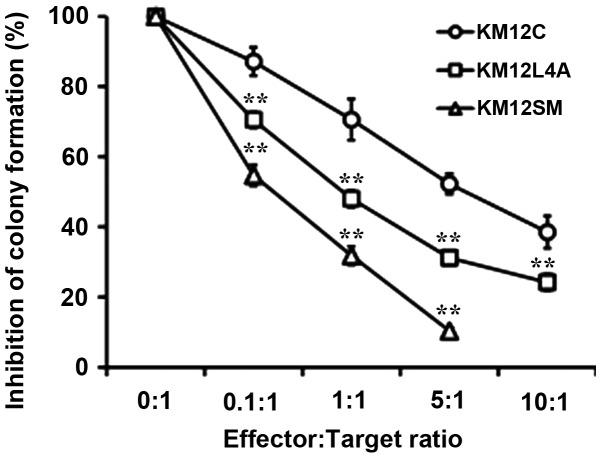
The susceptibility of (A) primary KM12C cells and highly metastatic cells (B) KM12L4A and (C) KM12SM to the natural killer cell line NK-92. Data, presented as mean ± standard error of three independent experiments, were analyzed by a Student’s *t*-test (^**^P<0.01).

The susceptibility to NK92 cells was compared between the primary KM12C cells and the metastatic sublines (Fig. 4). Although the clonogenicity of the KM12L4A and KM12SM cells was higher than that of KM12C cells, the susceptibility to NK92 cells of the KM12L4A and KM12SM cells was also significantly higher than that of KM12C cells. These results suggest that the metastatic cancer cell populations, which may contain a greater proportion of cancer stem-like cells, are not necessarily resistant to NK cell-mediated lysis.

## Discussion

Metastasis is responsible for ≤90% of mortalities from solid cancers (1,2), primarily due to its systemic nature and the resistance of disseminated tumor cells to existing anticancer therapies (3). As CSCs are thought to be responsible for metastasis and therapy-resistance (4), the current study investigated the levels of CSC markers, the clonogenicity in soft agar and the susceptibility to NK cells of metastatic colon cancer cells, including the primary KM12C cell line and the highly metastatic sublines, KM12SM and KM12L4A, which have previously been shown to be resistant to anticancer drugs and ionizing radiation (21).

Currently, few definitive stem cell markers are available for the investigation of CSCs in solid tumors. In colon cancers, certain putative cancer stem cell markers, including CD133, CD44 and EpCAM, have been used to identify colon CSCs (5,22). In the current study, a high percentage of KM12L4A and KM12SM cells were positive for all markers, including CD44, CD133 and EpCAM. The majority of KM12C cells were also positive for CD44 and EpCAM, however, only ~55% of these cells were positive for CD133. In addition, the mRNA levels of stemness genes, including SOX-2, Nanog and OCT-4 were higher in KM12L4A and KM12SM than in KM12C cells. Consistently, increased clonogenicity of the two highly metastatic sublines in soft agar was observed compared with KM12C cells. KM12SM and KM12L4A were derived from the parental, poorly metastatic cell line, KM12C, following injection into the cecum and spleen of nude mice to produce spontaneous and experimental hepatic metastases, respectively (15). KM12SM cells were selected for their growth in the cecum, invasion into the circulation, and survival and growth in the parenchyma of the liver; KM12L4A cells were selected for their survival in the circulation and survival and growth in the liver. Each subline exhibited a near tetraploid number of chromosomes, in contrast to the parental KM12C cells, which were near diploid with a minor tetraploid subpopulation (15,23). Additionally, a variety of metastasis-associated genes have been reported to be upregulated and downregulated in KM12SM and KM12L4A cells compared with in KM12C cells (24). We hypothesize that during the genetic evolution of KM12SM and KM12L4A cells, the stemness of these cells may increase due to an increase in the levels of stemness genes. These genes are associated with distant recurrence and poor disease-free survival in colorectal cancer following chemoradiotherapy (25), and colony-forming activity in soft agar, which is associated with the characteristics of cancer stem cells, which have been suggested to be the cell renewal source of a neoplasm and the seeds for the metastatic spread of cancer (20). Therefore, the increased levels of putative markers of CSCs and stemness genes may be associated with the increased clonogenicity of the highly metastatic KM12SM and KM12L4A cells, suggesting that highly metastatic colon cancer cell populations may contain a greater proportion of cancer stem-like cells.

CD133 was used in the initial studies on colon CSCs, revealing that CD133+ cells isolated from colorectal cancer exhibited the properties of self-renewal and high tumorigenic potential, compared with CD133*-* cells, which were unable to initiate tumor growth (26,27). It has been demonstrated that CD133 is associated with enhanced colony formation in 2D and 3D culture in colorectal cancer cells (28). In the present study, the highly metastatic KM12SM and KM12L4A cells, which exhibited higher levels of CD133, had greater clonogenicity compared with the poorly metastatic KM12C cells. However, the reliability of CD133 as a marker of colon CSCs is controversial as it has been demonstrated that CD133+ and CD133-metastatic tumor subpopulations formed colonospheres in *in vitro* cultures and were capable of long-term tumorigenesis in a NOD/SCID serial xenotransplantation model (29,30). Dalerba *et al* (31) demonstrated that the ability to engraft *in vivo* in immunodeficient mice was restricted to a minority subpopulation of CD44+ epithelial cells with high levels of EpCAM expression. In the current study, the majority of cells of the three KM12 series sublines were EpCAM+ and CD44+. Therefore, CSC markers other than CD133, CD44 and EpCAM may be necessary to identify CSCs in KM12 cell populations.

The loss of MHC molecules is often observed in advanced metastatic cancer cells, rendering tumor cells resistant to CD8+ T-cell-mediated cytotoxicity (32). The levels of NKG2D ligands (which can be recognized by other T-cell subsets, including γδ T cells and NK cells) (33) and of TRAIL receptors (which induce apoptosis in transformed cells but not in normal cells) (12) may therefore affect the susceptibility of the highly metastatic colon cancer cells to NK cells. In the present study, the levels of NKG2D ligands and DR4/5 were generally higher in the highly metastatic KM12L4A and KM12SM cells compared with that in the primary KM12C cells, and this result was consistent with the increased susceptibility to NK92 cells of the KM12L4A and KM12SM clonogenic cells compared with the KM12C clonogenic cells. However, the clonogenicity of KM12L4A and KM12SM cells was markedly higher than that of KM12C cells. NK cells are essential in the control of tumors with upregulated ligands for NK activation receptors and/or loss of MHC-I molecules (13). The NKG2D activation receptor binds to a group of ligands that includes MICA, MICB, and the family of ULBP molecules in humans; the expression of these molecules may be induced in cells under a variety of stresses including transformation, heat shock, oxidative stresses or DNA damage (34–37). High expression of MIC or RAET1G has been shown to be associated with prolonged survival of patients with colorectal tumors (38). It has also been demonstrated that activated NK cells with membrane-bound TRAIL enhance NK cell cytotoxicity against neuroblastoma cells (39). In addition, colorectal carcinoma-derived cancer-initiating cells (CICs) were more susceptible to freshly purified allogeneic NK cells than the non-CIC counterpart of the tumors, due to the higher expression of ligands for NKp30 and NKp44 in the natural cytotoxicity receptor group of activating NK receptors in CICs (40). Therefore, the results of the present study suggest that metastatic cancer cells, which may consist of a greater number of cancer stem-like cells, are not necessarily resistant to NK cell-mediated lysis, and the levels of NKG2D ligands and TRAIL receptors may affect the susceptibility of highly metastatic colon cancer cells to NK-mediated lysis. However, further studies using other metastatic cancer models are required to generalize this hypothesis.
